# Supplementary material for: A quantum optical study of thresholdless lasing features in high-β nitride nanobeam cavities
Source: Nat Commun. 2018 Feb 8;9:564. doi: 10.1038/s41467-018-02999-2 (PMC5805739; doi:10.1038/s41467-018-02999-2)
Supplement: Supplementary file 1 — Supplementary Information [file 41467_2018_2999_MOESM1_ESM.pdf]

A quantum optical study of thresholdless lasing features in high-β nitride nanobeam cavities

Jagsch et al.

Supplementary Note 1: Microscopic Laser Model

Our theoretical analysis comprises the input-output characteristics, the zero delay time second-order photon correlation function $g^{(2)}(\tau=0)$ and the coherence time obtained from a two-time calculation of the first-order autocorrelation function $g^{(1)}(t,\tau)$. The model accounts for quasi-continuous k-states of the two-dimensional quantum well (QW) gain material, the lowest-energy mode that is provided by the cavity and their mutual interaction. We use a quantized light field in order to access the statistical properties of the emission via $g^{(2)}(\tau=0)$ and to naturally include spontaneous emission in our model. The operators $b^\dagger$ and b create or annihilate a photon in the laser mode and operators v_k and c_k refer to carriers in the valence- and conduction-band states of the gain material. In this notation the Hamiltonian of the system is written as

$$H = H_{\mathrm{carr}} + H_{\mathrm{ph}} + H_{\mathrm{I}}, \tag{1}$$

where H_{carr} and H_{ph} are the Hamiltonians of the charge carriers and photons

$$H_{\mathrm{carr}} = \sum_k \varepsilon_k^{\mathrm{e}} c_k^\dagger c_k + \sum_k \varepsilon_k^{\mathrm{h}} v_k^\dagger v_k, \tag{2}$$

$$H_{\mathrm{ph}} = \hbar\omega\left(b^\dagger b + \tfrac{1}{2}\right), \tag{3}$$

and H_{I} is the interaction Hamiltonian

$$H_{\mathrm{I}} = \mathrm{i}\sum_k \left(g b v_k c_k^\dagger - g^* b^\dagger v_k^\dagger c_k\right). \tag{4}$$

Energies $\varepsilon_k^{\mathrm{e}}$ and $\varepsilon_k^{\mathrm{h}}$ are the electron and hole energies for different momenta k and $\hbar\omega$ is the photon energy of the cavity mode. For a solid quantum well, as it is found e.g. in a VCSEL structure, we define the light-matter coupling constant g as

$$g = \sum_{\mathbf{q}_\parallel} 2E_{\mathrm{ph}}\Gamma_z \boldsymbol{d}_{\mathrm{cv}}\tilde{\boldsymbol{u}}(\boldsymbol{q}_\parallel). \tag{5}$$

$E_{\mathrm{ph}} = \sqrt{\frac{\hbar\omega}{2\varepsilon_0 V_{\mathrm{res}}}}$ is the field per photon in the cavity mode with V_{res} the resonator volume, Γ_z the confinement factor in z-direction (vertical to the QW), $\boldsymbol{d}_{\mathrm{cv}}$ the dipole moment between valence and conduction band and $\tilde{\boldsymbol{u}}(\mathbf{q}_\parallel)$ the Fourier transform of the (cavity-) mode function inside the QW plane.

An approximate relation between the light-matter coupling constant and the spontaneous emission time can be established by adiabatically solving Eqn. (10) and inserting it into Eqns. (8) and (9) while neglecting all terms but the one corresponding to spontaneous emission into the lasing mode and only considering the k-value in spectral resonance with the optical mode. For k-values other than the ones close to resonance, the detuning reduces the light-matter interaction. One arrives at the expression

$$g = \sqrt{\frac{\kappa+\Gamma}{2\tau_\mathrm{l}}} \tag{6}$$

where κ is the inverse cavity lifetime, Γ is the dephasing rate and τ_l is the spontaneous emission time into the cavity.

In addition, dissipative processes enter the following equations of motion via Lindblad terms.

Coupled Laser Equations

Using Heisenberg's equation of motion and a truncation of the arising hierarchy of coupled equations along the lines of Ref. [1], we arrive at dynamical equations for the mean photon number $\langle b^\dagger b\rangle$ and the carrier-distribution functions $f_k^\mathrm{e}, f_k^\mathrm{h}$ (for electrons e and holes h separately),

$$\left(\hbar\frac{\mathrm{d}}{\mathrm{d}t} + 2\kappa\right)\langle b^\dagger b\rangle = 2|g|^2 \sum_{k'} \mathrm{Re}\left[\langle b^\dagger v_{k'}^\dagger c_{k'}\rangle\right], \tag{7}$$

$$\hbar\frac{\mathrm{d}}{\mathrm{d}t} f_k^\mathrm{e} = -2|g|^2 \mathrm{Re}\left[\langle b^\dagger v_k^\dagger c_k\rangle\right] - \gamma_\mathrm{nl} f_k^\mathrm{e} f_k^\mathrm{h} - \gamma_\mathrm{rel}^\mathrm{e}\left(f_k^\mathrm{e} - f_k^\mathrm{F.D.}\right) - A_\mathrm{nr} f_k^\mathrm{e} + P f_k^{(0)}\left(1 - f_k^\mathrm{e}\right), \tag{8}$$

$$\hbar\frac{\mathrm{d}}{\mathrm{d}t} f_k^\mathrm{h} = -2|g|^2 \mathrm{Re}\left[\langle b^\dagger v_k^\dagger c_k\rangle\right] - \gamma_\mathrm{nl} f_k^\mathrm{e} f_k^\mathrm{h} - \gamma_\mathrm{rel}^\mathrm{h}\left(f_k^\mathrm{h} - f_k^\mathrm{F.D.}\right) - A_\mathrm{nr} f_k^\mathrm{h} + P f_k^{(0)}\left(1 - f_k^\mathrm{h}\right), \tag{9}$$

where P is the pump rate. Their dynamics are governed by the photon-assisted polarization $\langle b^\dagger v_k^\dagger c_k\rangle$ that describes the process of photon emission via a carrier transition from a conduction-band to a valence-band state k. Photon losses expressed by the Q-factor of the mode are accounted for by the cavity loss rate $\kappa = E_\mathrm{ph}/Q$. The carrier dynamics is subject to both radiative and non-radiative losses at rates γ_nl and A_nr as well as pumping.

To simulate the experimental situation of carrier excitation in the barrier material the subsequent capture into the QW quasi-continuum states, we assume a carrier generation that is Gaussian distributed higher above the band edge from where carriers relax to the band edges. Carrier relaxation towards quasi-equilibrium is treated in terms of a relaxation-time approximation against respective Fermi-Dirac distributions $f_k^\mathrm{F.D.}$ for electrons and holes at rates $\gamma_\mathrm{rel}^\mathrm{e}$ and $\gamma_\mathrm{rel}^\mathrm{h}$. The carrier distribution functions $f_k^\mathrm{e}, f_k^\mathrm{h}$ that enter the theory are generally non-equilibrium distributions and reflect effects such as hole burning at the cavity-mode energy in the presence of stimulated emission, which is indicative for lasing and discussed in the context of Supplementary Figure 2 below.

In standard laser theory, radiative losses are typically associated with the β-factor, which is a measure for the fraction of the spontaneous emission directed into the laser mode. At the same time, the β-factor relates to the occurrence of a jump in the input-output curve. If radiative losses are small in comparison to emission into the laser mode, the β-factor can approach unity and the input-output curve becomes thresholdless. As radiative recombination requires the presence of an electron and a hole, radiative losses are proportional to $f_k^\mathrm{e} f_k^\mathrm{h}$. Non-

radiative losses, on the other hand, arise from different physical effects, such as Shockley-Read-Hall recombination or Auger processes. We use a general loss rate A_{nr} that is proportional to the respective carrier population functions f_k^{e} and f_k^{h} [2]. As a general property, non-radiative losses lead to an increased slope - larger than one - in the double logarithmic input-output curve in the low-excitation regime. Furthermore, it is worth noting that both, radiative and non-radiative losses, if sufficiently strong, give rise to a jump in the input-output curve and cannot easily be separated without further investigations.

The non-Markovian equation for the photon-assisted polarization is the central quantity that contains the light-matter interaction of the gain material with photons in the laser mode. Its equation of motion is given by

$$\begin{aligned}\left(\hbar\frac{\mathrm{d}}{\mathrm{d}t}+\kappa+\Gamma\right)\langle b^\dagger v_k^\dagger c_k\rangle &= -\mathrm{i}(\varepsilon_k^{\mathrm{e}}-\varepsilon_k^{\mathrm{h}}-\hbar\omega)\langle b^\dagger v_k^\dagger c_k\rangle \\ &+f_k^{\mathrm{e}}f_k^{\mathrm{h}}+\langle b^\dagger b\rangle(f_k^{\mathrm{e}}+f_k^{\mathrm{h}}-1)+\delta\langle b^\dagger b c_k^\dagger c_k\rangle-\delta\langle b^\dagger b v_k^\dagger v_k\rangle. \end{aligned} \tag{10}$$

The light-matter interaction Hamiltonian gives rise to spontaneous emission proportional to $f_k^{\mathrm{e}}f_k^{\mathrm{h}}$, and stimulated processes proportional to $\langle b^\dagger b\rangle(f_k^{\mathrm{e}}+f_k^{\mathrm{h}}-1)$ plus correlation terms due to carrier-photon correlation functions $\delta\langle b^\dagger b c_k^\dagger c_k\rangle$ and $\delta\langle b^\dagger b v_k^\dagger v_k\rangle$. Depending on the sign of the population term, it represents stimulated emission (gain) or absorption proportional to the mean intra-cavity photon number. The free evolution of this equation is governed by the detuning of each electronic transition $\varepsilon_k^{\mathrm{e}}-\varepsilon_k^{\mathrm{h}}$ to the cavity-mode energy $\hbar\omega$. The bandwidth of the interaction is determined by the broadening of the cavity-mode resonance (passive cavity Q-factor) and the linewidth of the gain material that we describe by the phenomenological constant Γ (representing the dephasing rate of the gain medium).

The carrier-photon correlation terms $\delta\langle b^\dagger b c_k^\dagger c_k\rangle$ and $\delta\langle b^\dagger b v_k^\dagger v_k\rangle$ can have a significant impact on the emission characteristics of nanolasers with strong light-matter interaction. Their calculation is a prerequisite to access the photon autocorrelation function

$$g^{(2)}(\tau=0)=2+\frac{\delta\langle b^\dagger b^\dagger b b\rangle}{\langle b^\dagger b\rangle^2},$$

which requires to include four additional equations:

$$\left(\hbar\frac{\mathrm{d}}{\mathrm{d}t}+4\kappa\right)\delta\langle b^\dagger b^\dagger b b\rangle = 4|g|^2\sum_{k'}\mathrm{Re}\left[\langle b^\dagger b^\dagger b v_{k'}^\dagger c_{k'}\rangle\right], \tag{11}$$

$$\begin{aligned}\left(\hbar\frac{\mathrm{d}}{\mathrm{d}t}+3\kappa+\Gamma\right)\delta\langle b^\dagger b^\dagger b v_k^\dagger c_k\rangle &= -\mathrm{i}(\varepsilon_k^{\mathrm{e}}-\varepsilon_k^{\mathrm{h}}-\hbar\omega_l)\delta\langle b^\dagger b^\dagger b v_k^\dagger c_k\rangle \\ &-2|g|^2\langle b^\dagger v_k^\dagger c_k\rangle^2-(1-f_k^{\mathrm{e}}-f_k^{\mathrm{h}})\delta\langle b^\dagger b^\dagger b b\rangle \\ &+2f_k^{\mathrm{h}}\delta\langle b^\dagger b c_k^\dagger c_k\rangle-2f_k^{\mathrm{e}}\delta\langle b^\dagger b v_k^\dagger v_k\rangle \\ &+2\langle b^\dagger b\rangle(\delta\langle b^\dagger b c_k^\dagger c_k\rangle-\delta\langle b^\dagger b v_k^\dagger v_k\rangle) \\ &-2\sum_{k'}\delta\langle b^\dagger b c_{k'}^\dagger v_{k'}^\dagger v_{k'} c_k\rangle+\sum_{k'}\delta\langle b^\dagger b^\dagger v_{k'}^\dagger v_k^\dagger c_{k'} c_k\rangle, \end{aligned} \tag{12}$$

$$\left(\hbar\frac{\mathrm{d}}{\mathrm{d}t}+2\kappa\right)\delta\langle b^\dagger b c_k^\dagger c_k\rangle = -2|g|^2\mathrm{Re}\left[\delta\langle b^\dagger b^\dagger b v_k^\dagger c_k\rangle + \sum_{k'}\delta\langle b^\dagger v_{k'}^\dagger c_k^\dagger c_{k'} c_k\rangle + \left(\langle b^\dagger b\rangle + f_k^{\mathrm{e}}\right)\langle b^\dagger v_k^\dagger c_k\rangle\right], \quad (13)$$

$$\left(\hbar\frac{\mathrm{d}}{\mathrm{d}t}+2\kappa\right)\delta\langle b^\dagger b v_k^\dagger v_k\rangle = 2|g|^2\mathrm{Re}\left[\delta\langle b^\dagger b^\dagger b v_k^\dagger c_k\rangle - \sum_{k'}\delta\langle b^\dagger c_{k'}^\dagger v_k^\dagger v_{k'} v_k\rangle + \left(\langle b^\dagger b\rangle + f_k^{\mathrm{h}}\right)\langle b^\dagger v_k^\dagger c_k\rangle\right]. \quad (14)$$

By truncating higher-order correlation functions (see Ref. [1]), Eqns. (7)-(14) form the closed system of coupled laser equations that are used for obtaining the results presented in the main text. We model the quasi-continuous k-states by using a discrete grid of ~350 equally spaced points, which leads to a system of about 1400 coupled equations.

Discussion of the β-factor

The β-factor plays a fundamental role in rate equation theories and is often considered as a central device characteristic. While in rate equations it directly enters as a parameter, the intricate physical processes that determine the behavior of the introduced laser model do not allow one to treat the impact of radiative carrier losses in terms of a single parameter. Nevertheless, a parameter that contains the same physical meaning, i.e. the ratio of the spontaneous emission into the laser mode to the total spontaneous emission can be calculated from our theory by evaluating

$$\beta = \frac{\sum_k \frac{2|g|^2}{\Gamma+\kappa}L(k) f_k^{\mathrm{e}} f_k^{\mathrm{h}}}{\sum_k \left(\frac{2|g|^2}{\Gamma+\kappa}L(k)+\gamma_{\mathrm{nl}}\right) f_k^{\mathrm{e}} f_k^{\mathrm{h}}}, \quad (15)$$

where $L(k)$ is a Lorentzian lineshape function. The expression is derived by adiabatically solving Eqn. (10) for the steady state and inserting it in Eqns. (8) and (9) where non-radiative losses are omitted. In the calculation of the emission into the laser mode, stimulated contributions are suppressed, so that the obtained β factor truly characterizes the spontaneous emission behavior. The β-factor of around 0.7 given in the main text is an upper estimate on the basis of expression (15).

From this procedure it becomes clearer that formally differentiating between radiative and non-radiative losses in defining an efficiency-factor such as β is rather artificial. Non-radiative losses deplete the carrier populations in Eqns. (8) and (9) in a similar way to radiative losses $\propto \gamma_{\mathrm{nl}}$, and an alternative definition of the β factor can be formulated

$$\tilde{\beta} = \frac{\sum_k \frac{2|g|^2}{\Gamma+\kappa}L(k) f_k^{\mathrm{e}} f_k^{\mathrm{h}}}{\sum_k \left(\frac{2|g|^2}{\Gamma+\kappa}L(k)+\gamma_{\mathrm{nl}}+A_{nr}\right) f_k^{\mathrm{e}} f_k^{\mathrm{h}}}, \quad (16)$$

in order to characterize the overall efficiency of carrier recombination into the laser mode. With this discussion we aim at clarifying the role of β, which is very well defined in rate equation theories such as in Ref. [3], but less so in microscopic theories that do not depend on β, but allow for a definition of such a factor in one way or another.

Coherence Time

The coherence time is defined as

$$\tau_{\mathrm{c}} = \int_{-\infty}^{\infty} \frac{\left|g^{(1)}(t,\tau)\right|^2}{\left|g^{(1)}(t,0)\right|^2}\,\mathrm{d}\tau, \tag{17}$$

which requires calculating the τ-dynamics of the two-time first-order photon correlation function

$$g^{(1)}(t,\tau) = \frac{\langle b^\dagger(t)b(t+\tau)\rangle}{\langle b^\dagger(t)b(t)\rangle}. \tag{18}$$

For continuous-wave excitation, the first time argument corresponds to the steady-state time. The τ-dynamics is obtained by formulating equations of motion with derivatives taken with respect to the delay-time τ:

$$\hbar\frac{\mathrm{d}}{\mathrm{d}\tau}G(\tau) = \sum_k g^* P_k(\tau) - (\kappa + \mathrm{i}\hbar\omega)G(\tau), \tag{19}$$

$$\hbar\frac{\mathrm{d}}{\mathrm{d}\tau}P_k(\tau) = g(f_k^{\mathrm{c}} - f_k^{\mathrm{v}})G(\tau) - (\Gamma + \mathrm{i}(\varepsilon_k^{\mathrm{e}} - \varepsilon_k^{\mathrm{h}}))P_k(\tau). \tag{20}$$

Here, the abbreviations

$$G(\tau) = \langle b^\dagger(t)b(t+\tau)\rangle, \tag{21}$$

$$P_k(\tau) = \langle b^\dagger(t)v_k^\dagger(t+\tau)c_k(t+\tau)\rangle \tag{22}$$

are used. The initial conditions that enter the time integration of the equations are obtained from the steady-state values of the single-t time dynamics [4].

Choice of Parameters

For the unloaded cavity Q-factor in the absence of an absorptive gain material we use $Q = 2200$ for the lasing and $Q = 1800$ for the non-lasing nanobeam cavity based on the measurements close to transparency of the gain medium. In all cases, a spontaneous emission time of τ_{sp} = 5 ns is assumed [5], which leads to a calculated light-matter interaction constant of $g = 0.041\ \mathrm{ps}^{-1}$ at resonance according to Eqn. (6). The cavity loss rates for the investigated nanobeam cavities are $\kappa = 0.94\ \mathrm{ps}^{-1}$ ($Q = 2200$) and $\kappa = 1.15\ \mathrm{ps}^{-1}$ ($Q = 1800$), respectively, and the phenomenological dephasing is $\Gamma = 20\ \mathrm{ps}^{-1}$. For the quantum-well gain material, we estimate an effective area of 0.31 μm^2 from the 3D-FDTD simulations.

Radiative losses are small in the nanobeam geometry, which is known to strongly funnel photons into the guided laser mode and suppress emission into non-lasing modes. Defect-induced non-radiative losses lead to a deviation of the unity-slope in the input-output curve in the low excitation regime. At 300 K we assume $A_{\mathrm{nr}} = 5 \times 10^7 \mathrm{s}^{-1}$, and an absence of non-radiative losses at 156 K. These parameter choices are supported by the measurements,

as a steeper slope than unity is observed at room temperature (especially in Fig. 2d), whereas the 156 K input-output curve is almost flat with a slope of one. The set of parameters is summarized in Supplementary Table 1.

Supplementary Table 1 | Parameters used in the calculation

	LASING NANOBEAM (RT)	LASING NANOBEAM (156 K)	REFERENCE NANOBEAM (RT)
$\boldsymbol{Q}$	2200	2200	1800
$\boldsymbol{\tau_{sp}}$ (ns)	5	5	5
$\boldsymbol{\beta}$	0.7	0.7	0.7
$\boldsymbol{A_{nr}}$ (s^{-1})	5×10^7	0.0	5×10^7
$\boldsymbol{g}$ (ps^{-1})	0.041	0.041	0.041

Additional indicators for lasing in the nanobeam structure

In Supplementary Figure 1 we show the results for the coherence time that we obtain for the nanobeam laser and the non-lasing reference structure. While the low-excitation value is mainly determined by the cavity-Q factors, the increase in coherence time is indicative for the build-up of coherence in the system. While the nanolaser reaches coherence-time values is the ns range which is typical for above-threshold operation, the coherence time of the reference structure saturates at a value < 100 ps, indicating that fully coherent emission is not reached.

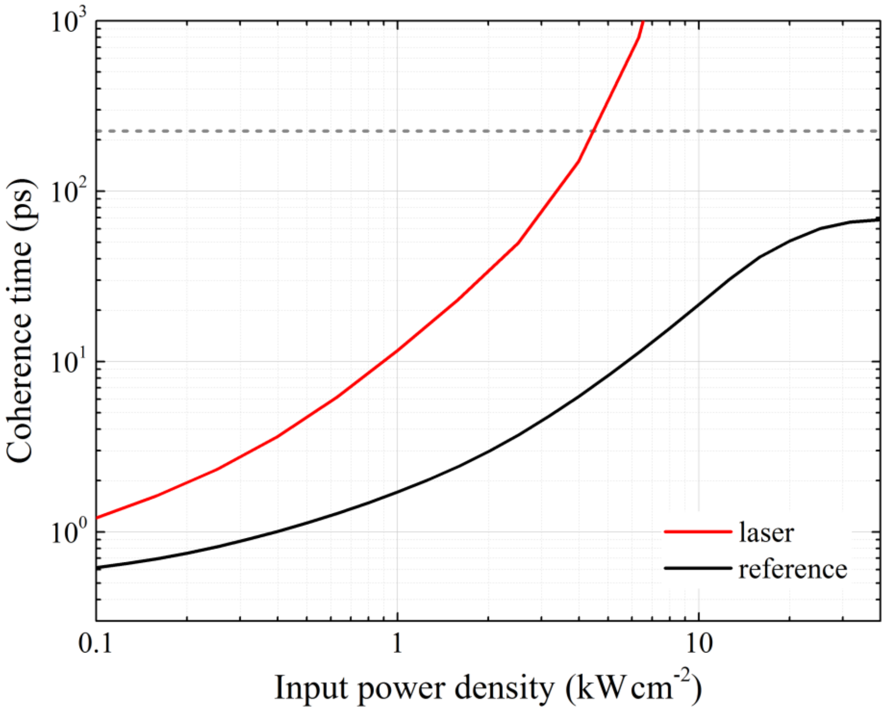

Supplementary Figure 1 | Calculated excitation-power-density dependent coherence time. Excitation dependent coherence time for the nanobeam laser and reference nanobeam according to Eqn. (17). The obtained coherence times are used when convoluting the ideal zero time delay autocorrelation function with the time resolution

of the HBT setup (225 ps – shown above with a dashed line) in order to simulate the experimental results.

The coherence time is of particular relevance to predict the measured autocorrelation function (dashed line in Figs. 2c and f in the main text) from the unconvoluted result (solid line in Fig. 2c and f in the main text), which would be obtained only with unlimited detector resolution. The dashed line in Supplementary Figure 1 indicates the experimental detector resolution (225 ps). If the coherence time is lower than the detector resolution, the measured autocorrelation function exhibits values that are closer to 1 than the true value.

From Eqns. (7)-(9) we obtain the wave-vector dependent carrier population functions for electrons and holes. In Supplementary Figure 2 we show results for the room temperature case corresponding to the data shown in Fig. 2 in the main text. Spectral hole burning (indicated by the shaded region in Supplementary Figure 2a) gives additional proof for the presence of stimulated emission in the nanolaser in the high-excitation regime and the absence of the same in the reference structure.

In general, the unambiguous identification of lasing in high-β nanocavity devices relies on a combination of indicators. The theoretical results for the coherence-time increase and the spectral hole burning in the population functions give valuable additional proof for the presence of lasing in addition to the experimental results that are shown in the main text.

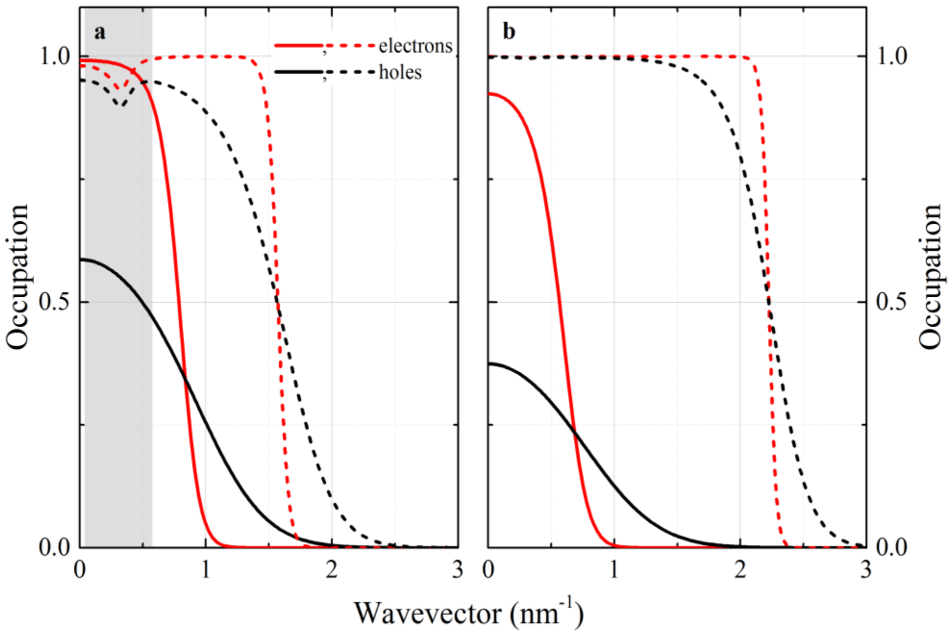

Supplementary Figure 2 | Carrier population functions and spectral hole burning. The non-equilibrium electron (red) and hole (black) distribution functions are shown for low (solid line) and high (dashed line) excitation power density. **a,** The nanobeam laser exhibits spectral hole burning at the laser-mode energy at high excitation (shaded region in **a**), which is indicative for lasing. **b,** No spectral hole burning is observed for the reference structure, confirming that it operates below threshold.

Supplementary Note 2: Investigation of sample heating

Heating of the nanobeam cavity was investigated by means of Raman thermometry and thermal transport simulations using COMSOL Multiphysics. For the Raman thermometry measurements the sample was excited under the same conditions as during the room temperature lasing characterization (methods section), using a 325 nm ultrasteep dielectric edge filter. Spectra were calibrated using a mercury gas discharge lamp and the spectrometer was not moved between measurements. The temperature increase in the cavity region was then inferred from the characteristic redshift of the polar $A_1(LO)$ phonon peak of the GaN matrix material [7]. We chose the $A_1(LO)$ phonon as a temperature sensor, as it is the most prominent Raman mode under the given resonant pumping conditions. An influence of the induced carrier density on the $A_1(LO)$ phonon (which would manifest itself in a blueshift of the resulting longitudinal phonon plasmon mode – the so-called LPP^+ mode) can be excluded [8-9]. Supplementary Figure 3 shows the excitation power density dependent shift of the GaN $A_1(LO)$ mode, as well as the shift of the dominating first-order mode of silicon, indicating the temperature of the illuminated region of the substrate, for comparison. In linear approximation the Raman shift RS of the two peaks is given as $\mathrm{d}T/\mathrm{dRS}(\mathrm{GaN}\ A_1(LO)) = -39\ \mathrm{K\ per\ cm^{-1}}$ and $\mathrm{d}T/\mathrm{dRS}(\mathrm{silicon}) = -46\ \mathrm{K\ per\ cm^{-1}}$ near room temperature [7], [10]. From the shifts in Supplementary Figure 3, the temperature of the cavity region increases by ~56 K, whereas the silicon substrate shows only a minor increase in temperature of ~8 K.

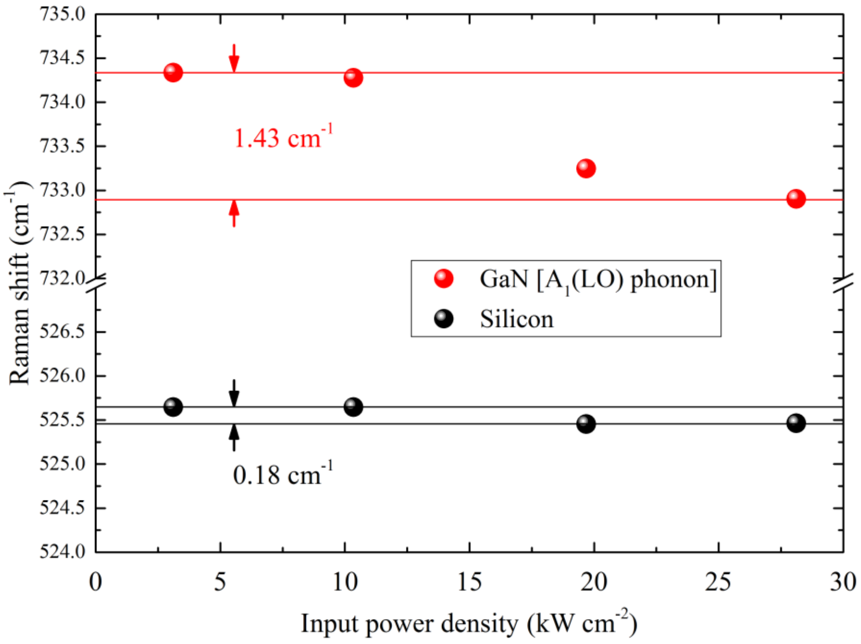

Supplementary Figure 3 | Raman thermometry measurements. Excitation power density dependent shift of the GaN $A_1(LO)$ and the first-order mode of silicon. From the overall redshift of the phonon modes, the temperature increase of cavity and substrate can be estimated to 56 K and 8 K, respectively.

Accompanying thermal transport simulations were carried out using COMSOL Multiphysics. For the simulations, a heat source was placed in the cavity region and the resulting temperature distribution along the nanobeam was subsequently simulated. Supplementary Figure 4 shows the temperature distribution (shown for half the

nanobeam) for several input powers. Solid and dashed lines correspond to a nanobeam with and without holes, respectively, indicating the lowered effective thermal conductivity in the actual structure. We can estimate an input power of ~200-300 μW (taking into account excitation density, illuminated cavity area and absorption coefficient) in the high excitation range during the characterization of the nanobeams, which results in an increase in cavity temperature of about 50-80 K in good agreement with the Raman thermometry. Note that the Raman measurements naturally involve an averaging over the laser spot size, which comprises a gradient for the excitation power density.

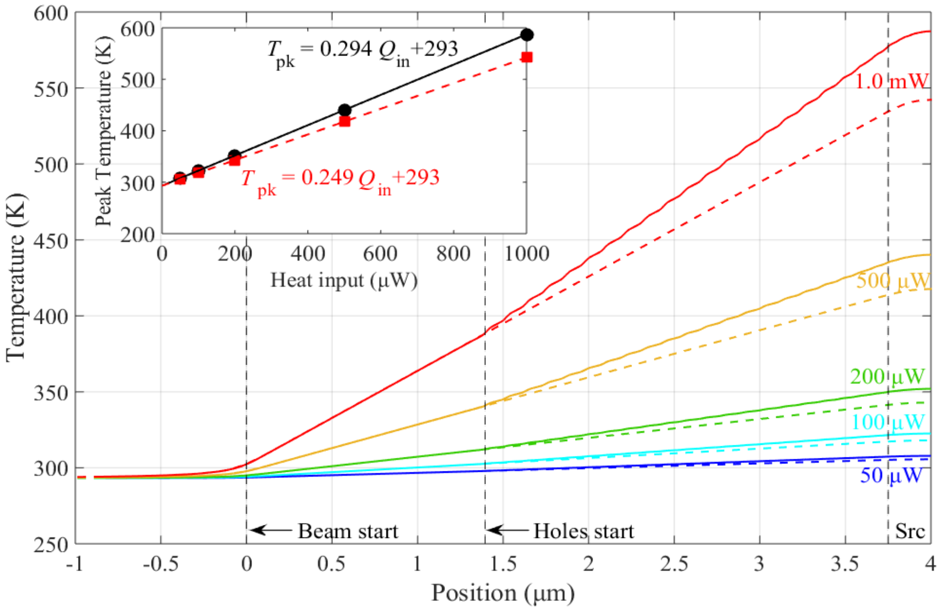

Supplementary Figure 4 | Thermal transport simulations. Temperature distribution along one half of the nanobeam (symmetric) for several input powers. Solid and dashed lines represent a nanobeam with and without holes, respectively. Inset: Dependence of the peak temperature T_{pk} on the heat input Q_{in}.

Supplementary Note 3: Evaluation of the intensity autocorrelation function

The measured autocorrelation traces were fitted using a convolution of the idealized fitting function $\tilde{g}^{(2)}(\tau) = 1 + g_0 \exp(-2|\tau|/\tau_{\text{cor}})$, with correlation time τ_{cor} and bunching amplitude g_0, and the detector response (assumed Gaussian) [6]

$$g^{(2)}(\tau) = \frac{1}{\sigma\sqrt{2\pi}} \int_{-\infty}^{\infty} \tilde{g}^{(2)}(\tau - \tau') \exp\left(\frac{-\tau'^2}{2\sigma^2}\right) d\tau', \quad (23)$$

where $\sigma = \Delta t_{\text{res}}/2\sqrt{2\ln(2)}$. The temporal resolution of the HBT setup is $\Delta t_{\text{res}} \approx 225$ ps. Supplementary Figure 5a shows the unbinned data (gray) at an excitation power density of 6.77 kW cm^{-2}, as well as the data using an 8x binning (black) overlaid with the convoluted fit to the unbinned data (red). Supplementary Figure 5b shows the evolution of $g^{(2)}(\tau)$ for six excitation power densities around threshold. At $\tau = 0$ we observe a clear bunching behavior ($g^{(2)}(0) > 1$), which becomes less pronounced with increasing excitation power density, indicating the transition from thermal light to coherent light.

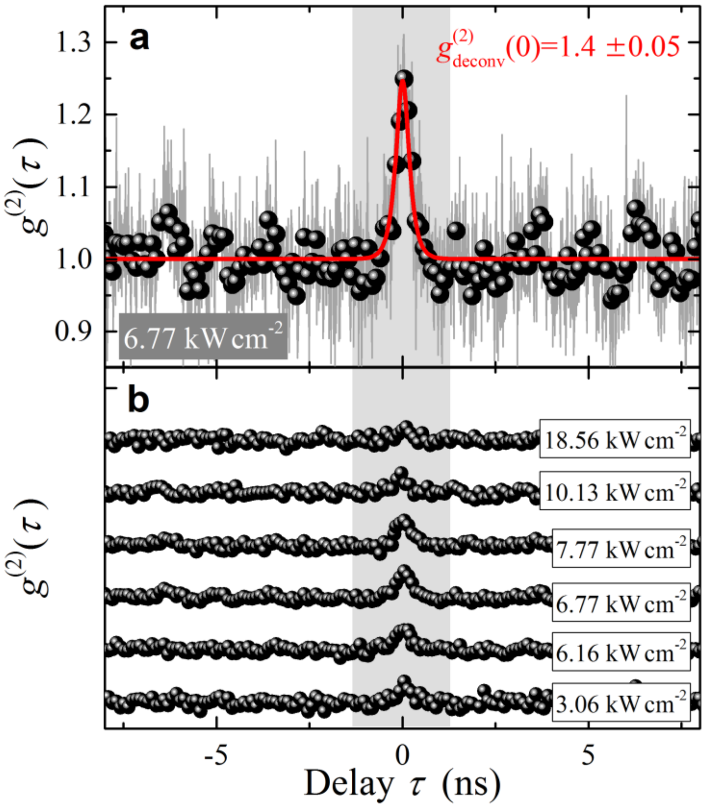

Supplementary Figure 5 | Room temperature second-order autocorrelation measurements of the nanobeam emission. a, Exemplary $g^{(2)}(\tau)$-trace with convoluted fit, Eqn. (23), to the unbinned data (gray) and the data using an 8x binning (black) for clarity. A deconvolved zero delay time value of $g^{(2)}_{\mathrm{deconv}}(0) = 1.4 \pm 0.05$ is extracted. **b,** Excitation power density dependent evolution of the second-order autocorrelation function (offset for clarity). As the excitation power density is increased across the threshold region, thermal bunching around $\tau = 0$ (shaded region) becomes more pronounced, before vanishing again at higher excitation power densities as the emission becomes increasingly coherent.

Supplementary Note 4: I-O behavior at low temperatures and impact of localized states due to indium composition fluctuations in the single quantum well

We observe a vanishing kink in the I-O curve of the nanobeam laser as the temperature is decreased below room temperature, until the device exhibits thresholdless (linear) I-O behavior despite a cavity $\beta < 1$ and the presence of non-radiative losses. Further insight into the lasing characteristics and the mechanism leading to a thresholdless intensity curve is gained via additional temperature dependent measurements below 156 K. Upon decreasing sample temperature, the I-O curve develops an inverse s-shape with an increased output intensity in the low excitation regime (cf. Fig. 3e of the main text), which we observed down to 20 K (not shown). In contrast, a true ($\beta = 1$) thresholdless device requires vanishing non-radiative losses and is expected to remain thresholdless once non-radiative recombinations become negligible. We attribute the I-O temperature dependence to indium composition fluctuations in the InGaN single quantum well (SQW) that lead to a

subsystem of localized states [11-12], thereby constituting a 0D-2D two component gain medium. The impact of such localized states has been thoroughly studied for InGaN quantum wells grown on other substrates [11-14]. A typical indicator for the presence of localized states is a blueshift of the QW emission peak energy at intermediate temperatures, following an initial redshift at low temperatures instead of the usual Varshni shift [13-14].

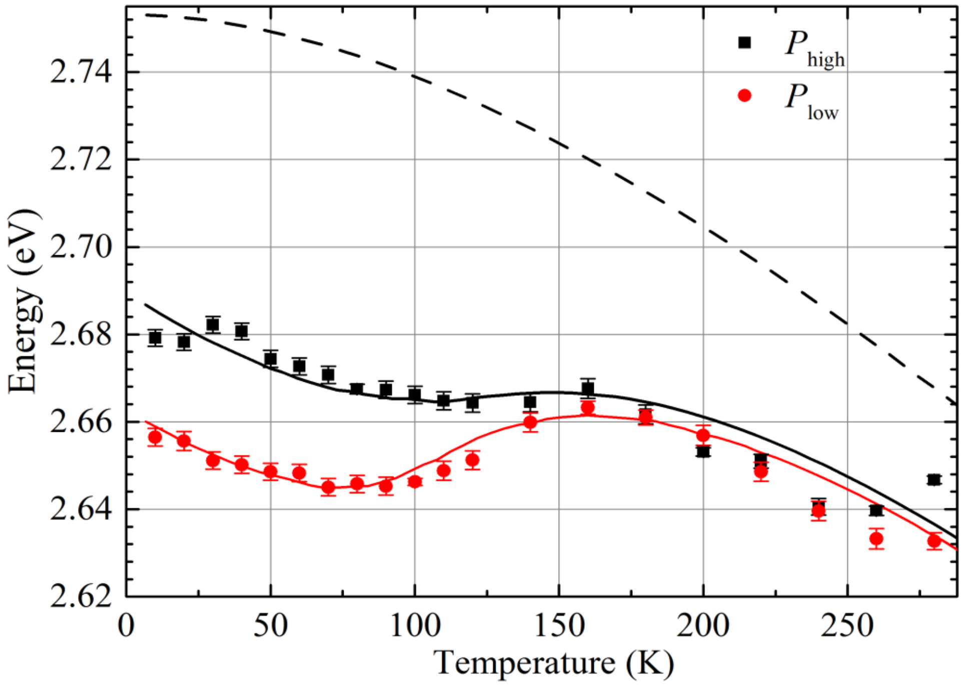

Supplementary Figure 6 | Temperature dependence of the InGaN/GaN SQW photoluminescence (PL). Peak energy extracted from InGaN SQW PL spectra for an unprocessed location on the sample obtained for two excitation power densities, namely $P_{\rm low} = 2.5\ \rm W\,cm^{-2}$ (red dots) and $P_{\rm high} = 51\ \rm W\,cm^{-2}$ (black dots). The blueshift occurring for temperatures above 90-110 K indicates the influence of localized states on the SQW emission. Fits (solid lines) accounting for the temperature dependence of the measured PL peak energy are issued from Eqns. (3) and (4) in Ref. [15] whereas the black dashed line corresponds to the well-known Varshni's empirical formula. Error bars indicate the standard deviation obtained from fitting the recorded spectra.

Supplementary Figure 6 shows the QW emission peak energy for two input power densities as a function of temperature for an unprocessed location on the sample up to $T = 280$ K, capturing the initial red shift and the subsequent blue shift due to localization starting at 90-110 K. The measurements were performed in standard (macro) PL configuration in the low excitation regime, using the 325 nm line of a HeCd laser at an input power density of 2.5 and 51 $\rm W\,cm^{-2}$, respectively. To decrease the inherent light waveguiding originating from the sample geometry, the sample was capped by a $\lambda/4n$ thick ($\sim$ 78 nm) SiO_2 layer tuned to the QW emission wavelength in order to promote vertical light extraction and hence observe QW PL emission up to 280 K in the low excitation regime. The temperature dependence of the measured PL peak energy can be well accounted for by using the model introduced by Li and co-workers [15] that considers a Gaussian-like distribution of localized electronic states. Within this model the QW emission energy is given by:

$$E_{\mathrm{QW}} = E_0 - \frac{\alpha_{\mathrm{V}} T^2}{\beta_{\mathrm{V}} + T} - x(T) k_{\mathrm{B}} T, \tag{24}$$

where E_0 is the free QW exciton energy at 0 K, α_{V} and β_{V} are the usual parameters entering in Varshni's empirical formula, k_{B} is the Boltzmann constant and $x(T)$ is a dimensionless coefficient obtained when solving the transcendental equation:

$$x \exp x = \left(\frac{\tau_{\mathrm{r}}}{\tau_{\mathrm{tr}}}\right) \left[\left(\frac{\sigma'}{k_{\mathrm{B}} T}\right)^2 - x\right] \exp\left[\frac{(E_0 - E_{\mathrm{a}})}{k_{\mathrm{B}} T}\right], \tag{25}$$

where τ_{r} is the carrier recombination time, τ_{tr} is the carrier transfer time between localized states, σ' is the standard deviation of the Gaussian-like distribution of localized electronic states and the energy difference $E_{\mathrm{a}} - E_0$ describes the magnitude of carrier localization at 0 K.

The results of the fitting procedure are summarized hereafter. An energy of $E_0 = 2.753$ eV is deduced for both investigated input power densities. The fact that the measured emission peak energies remain the same for temperatures larger than $\sim$160 K, within the error margin of the measurements, indicates that no screening of the built-in field due to injected carriers occurs. This behaviour is fully consistent with the low cw input power densities. Hence the reported emission peak energy blueshift for low temperatures ($T <$ 160 K) between the two input power densities can be ascribed to a progressive filling of localized states. σ' amounts to 28 ± 0.5 meV for both power densities whereas values of 89 meV and 62 meV are extracted for the energy difference $E_{\mathrm{a}} - E_0$ for the lower and the higher excitation power density, respectively. Additional insights about the bare SQW emission properties can be deduced from the temperature dependence of the integrated PL intensity for the two above-mentioned input power densities (Supplementary Figure 7). Thus the larger integrated PL intensity measured for low temperatures for the higher input power density case can be satisfactorily explained in the framework of a faster carrier transfer time combined with a shorter radiative carrier recombination time. The nearly identical value extracted for the thermal activation energy $E_{\mathrm{A}} =$ 80-84 meV for the two investigated input power densities is one more signature that carriers get delocalized above a certain temperature and that they likely experience the same 2D-like potential as also corroborated by their similar emission peak energy.

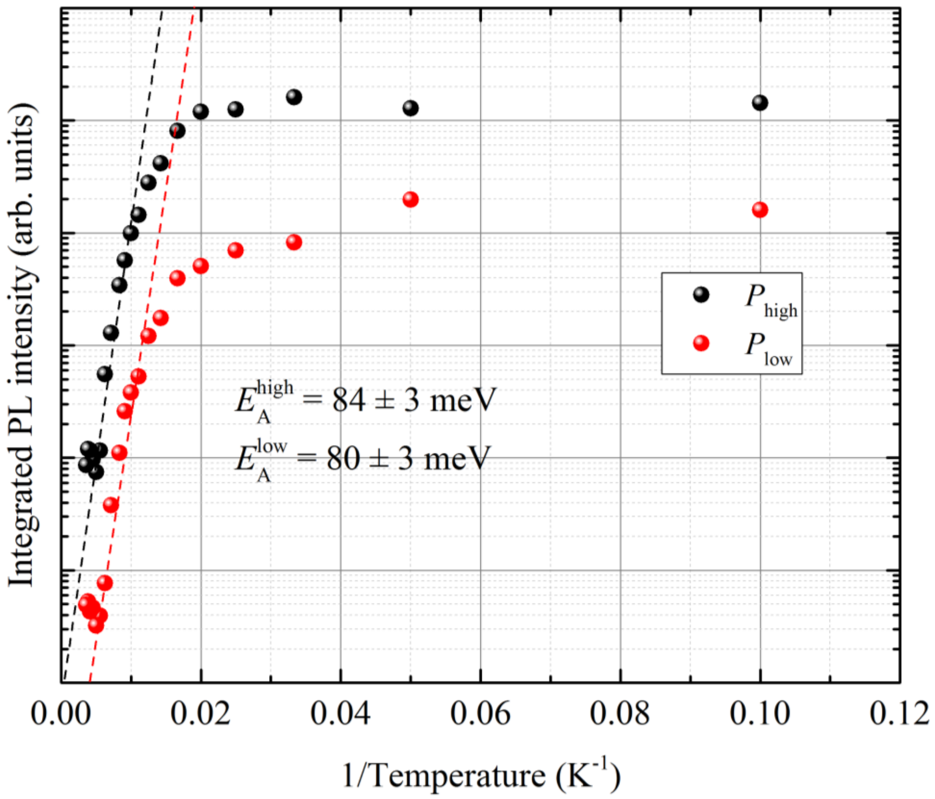

Supplementary Figure 7 | Temperature dependence of the integrated PL intensity of the InGaN/GaN SQW. Temperature dependence of the integrated PL intensity of the bare InGaN/GaN SQW sample obtained for two input power densities, namely $P_{\text{low}} = 2.5\ \text{W cm}^{-2}$ (red dots) and $P_{\text{high}} = 51\ \text{W cm}^{-2}$ (black dots) together with the corresponding high temperature ($T > 160$ K) thermal activation energy.

When transferring the SQW emission features to the case of the nanobeam laser, at low excitation, 0D-like states are most certainly the main contribution to the gain and the excitation power dependence is linear. As the excitation power density increases, the 0D subsystem saturates and the 2D component increasingly dominates, resulting in an inverse s-shaped I-O curve as a result of the transition from one subsystem to the other. In an intermediate temperature range around 150 K non-radiative losses are exactly compensated by this gain transition, leading to the observed thresholdless I-O curve. We therefore conclude that a thresholdless behavior can be mimicked by a complex gain medium, even in case of a non-ideal spontaneous emission coupling (i.e. $\beta < 1$). Importantly, this effect can lead to an incorrect interpretation of close to linear I-O curves when studying nanolasers only at a single temperature. This section clearly evidences the need for quantum optical experiments, such as power dependent second-order autocorrelation measurements, as well as a temperature dependent investigation in order to unambiguously assess the transition from spontaneous to stimulated emission and to prove thresholdless behavior in high-β nanolasers.

Supplementary Note 5: Optical images of the emission

Supplementary Figure 8 shows optical images of the nanobeam emission that have been collected for different excitation power densities. Excitation laser and GaN emission have been filtered out using an appropriate low-pass filter. Background emission from the QW gain material is collected as well. With increasing excitation power density we observe enhanced directionality of the emission perpendicular to the nanobeam, in agreement with [16]. The appearance of a 'stripe pattern' (cf. detail in Supplementary Figure 8) is attributed to the structure itself. We would like to note in this context that the appearance of speckles, fringes or generally interference effects in optical images of a device's emission cannot be seen as (conclusive) evidence for lasing. They are a field coherence effect (first-order coherence) and can also be produced by passing light through a narrow bandpass [17] or spatial filter (e.g. double slit experiment by Thomas Young).

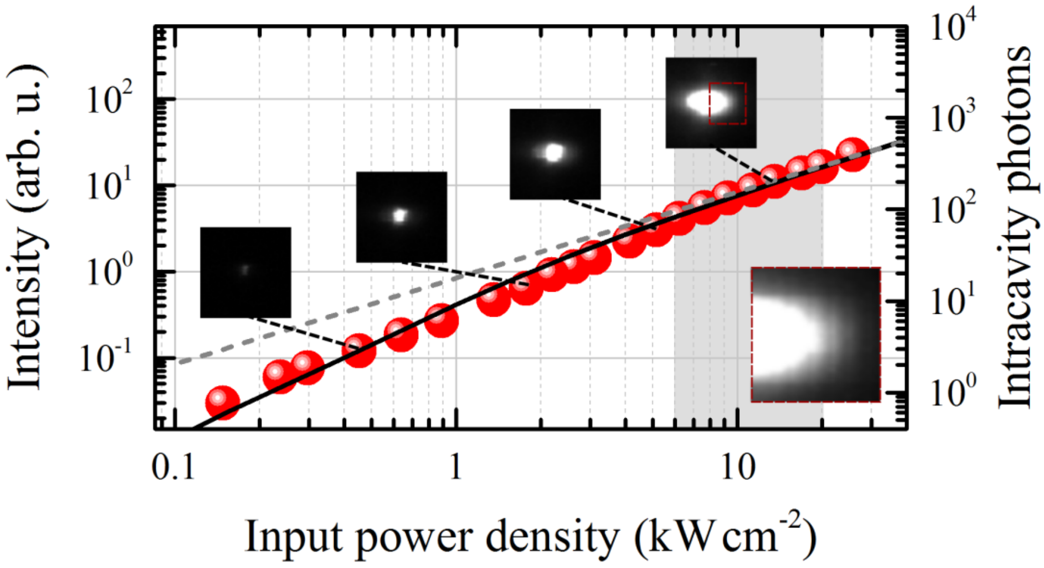

Supplementary Figure 8 | Optical images of the nanobeam emission. Room temperature I-O curve (panel **a** of Fig. 2 in the main text) along with optical images of the emission. We observe an increased directionality of the emission with increasing excitation. Detail: We attribute the appearance of a slight 'stripe pattern' parallel to the nanobeam to the structure itself.

Supplementary References

[1] Gies, C., Wiersig, J., Lorke, M. & Jahnke, F. Semiconductor model for quantum-dot-based microcavity lasers. *Phys. Rev. A* **75**, 013803 (2007)

[2] Chow, W.W., Jahnke, F. & Gies, C. Emission properties of nanolasers during the transition to lasing. *Light-Sci. Appl.* **3,** e201 (2014).

[3] Yokoyama, H. & Brorson, S. D. Rate equation analysis of microcavity lasers. *J. Appl. Phys.* **66,** 4801-4805 (1989).

[4] Ates, S., Gies, C., Ulrich, S. M., Wiersig, J., Reitzenstein, S., Löffler, A., Forchel, A., Jahnke, F., & Michler, P. Influence of the spontaneous optical emission factor β on the first-order coherence of a semiconductor microcavity laser. *Phys. Rev. B* **78,** 155319 (2008).

[5] Rossbach, G., et al. High-temperature Mott-transition in wide band-gap semiconductor quantum wells. *Phys. Rev. B* **90,** 201308 (2014). Supplementary Material.

[6] Ulrich, S. M. et al. Photon Statistics of Semiconductor Microcavity Lasers. *Phys. Rev. Lett.* **98,** 043906 (2007).

[7] Li, W. S., Shen, Z. X., Feng, Z. C., Chua, S. J. Temperature dependence of Raman scattering in hexagonal gallium nitride films. *J. Appl. Phys.* **87,** 3332-3337 (2000).

[8] Haboeck, U., Siegle, H., Hoffmann, A., Thomsen, C. Lattice dynamics in GaN and AlN probed with first- and second-order Raman spectroscopy. *phys. stat. sol. c.* **0,** 1710-1731 (2003).

[9] Kuball, M. Raman spectroscopy of GaN, AlGaN and AlN for process and growth monitoring/control. *Surf. Interface Anal.* **31,** 987-999 (2001).

[10] Balkanski, M., Wallis, R. F., Haro, E., Anharmonic effects in light scattering due to optical phonons in silicon. *Phys. Rev. B.* **28,** 1928-1934 (1983).

[11] Chichibu, S. F. et al. Origin of defect-insensitive emission probability in In-containing (Al,In,Ga)N alloy semiconductors. *Nat. Mater.* **5,** 810-816 (2006).

[12] Schulz, T., Remmele, T., Markurt, T., Korytov, M. & Albrecht, M. Analysis of statistical compositional alloy fluctuations in InGaN from aberration corrected transmission electron microscopy image series. *J. Appl. Phys.* **112,** 033106 (2012).

[13] Cho, Y.-H., et al. "S-shaped" temperature-dependent emission shift and carrier dynamics in InGaN/GaN multiple quantum wells. *Appl. Phys. Lett.* **73,** 1370-1372 (1998).

[14] Feng, S.-W. et al. Impact of localized states on the recombination dynamics in InGaN/GaN quantum well structures. *J. Appl. Phys.* **92,** 4441-4448 (2002).

[15] Li, Q. et al. Thermal redistribution of localized excitons and its effect on the luminescence band in InGaN ternary alloys. *Appl. Phys. Lett.* **79,** 1810-1812 (2001).

[16] Rousseau, I., Sánchez-Arribas, I., Carlin, J.-F., Butté, R. & Grandjean, N. Far-field coupling in nanobeam photonic crystal cavities. *Appl. Phys. Lett.* **108,** 201104 (2016).

[17] Hayenga, W. E. et al. Second-order coherence properties of metallic nanolasers. *Optica* **3,** 1187-1193 (2016).
